# Optimization of process parameters in preparation of tocotrienol-rich red palm oil-based nanoemulsion stabilized by Tween80-Span 80 using response surface methodology

**DOI:** 10.1371/journal.pone.0202771

**Published:** 2018-08-24

**Authors:** Wai-Ting Chong, Chin-Ping Tan, Yoke-Kqueen Cheah, Ahmad Firdaus B. Lajis, Noor Lida Habi Mat Dian, Sivaruby Kanagaratnam, Oi-Ming Lai

**Affiliations:** 1 Institute of Bioscience, Universiti Putra Malaysia, Serdang, Selangor, Malaysia; 2 Department of Food Technology, Faculty of Food Science and Technology, Universiti Putra Malaysia, Serdang, Selangor, Malaysia; 3 Department of Biomedical Sciences, Faculty of Medicine and Health Sciences, Universiti Putra Malaysia, Selangor, Malaysia; 4 Protein and Food Technology Unit, Malaysian Palm Oil Board, Kajang, Selangor, Malaysia; 5 Department of Bioprocess Technology, Faculty Biotechnology and Biomolecular Sciences, Universiti Putra Malaysia, Serdang, Selangor, Malaysia; Clemson University, UNITED STATES

## Abstract

Red palm oil (RPO) is a natural source of Vitamin E (70–80% tocotrienol). It is a potent natural antioxidant that can be used in skin-care products. Its antioxidant property protects skin from inflammation and aging. In our work, a tocotrienol-rich RPO-based nanoemulsion formulation was optimized using response surface methodology (RSM) and formulated using high pressure homogenizer. Effect of the concentration of three independent variables [surfactant (5–15 wt%), co-solvent (10–30 wt%) and homogenization pressure (500–700 bar)] toward two response variables (droplet size, polydispersity index) was studied using central composite design (CCD) coupled to RSM. RSM analysis showed that the experimental data could be fitted into a second-order polynomial model and the coefficients of multiple determination (R^2^) is 0.9115. The optimized formulation of RPO-based nanoemulsion consisted of 6.09 wt% mixed surfactant [Tween 80/Span 80 (63:37, wt)], 20 wt% glycerol as a co-solvent via homogenization pressure (500 bar). The optimized tocotrienol-rich RPO-based nanoemulsion response values for droplet size and polydispersity index were 119.49nm and 0.286, respectively. The actual values of the formulated nanoemulsion were in good agreement with the predicted values obtained from RSM, thus the optimized compositions have the potential to be used as a nanoemulsion for cosmetic formulations.

## Introduction

Of late, consumer preference for natural ingredients has been increasing in both food and pharmaceutical industries. This has resulted in a significant increase in demand for the substitution of synthetic compounds by natural substances. Red palm oil (RPO) is one of the natural substances that has been shown to be a functional ingredient. Unlike normal refined, bleached and deodorised (RBD) palm oil, RPO is cold pressed and/or followed by deacidification and deodorisation using molecular distillation from the fruit of the oil palm tree, *Elaeisguineensis*[1,2]. The process of refining, bleaching and deodorising in RBD palm oil results in the removal of some vitamin E and the elimination of the carotenoids in the oil[3]. Conversely, RPO retains relatively higher amounts of carotenoids (500–700 ppm) and vitamin E (500–1000 ppm; tocopherols and tocotrienols) compared with RBD palm oil (350–630 ppm)[1–5]. Additionally, many recent studies had reported beneficial health properties of RPO. For instance, RPO has been demonstrated to improve vitamin A status, lipid profile and health disorders such as carotid atherosclerosis [6–8].

Over the past few decades, the growing demand for skin care products has resulted in the rapid growth of the global cosmetics market. Up till now, many cosmetics and dermatological products make use of vitamin E as part of their bioactive compounds component [9,10]. Vitamin E is a powerful lipophillic antioxidant and is proven to effectively treat dermatological problems such as melasma and skin inflammation [9,11,12]. For industry use, vitamin E in the form of tocopheryl acetate is chemically synthesized via cyclocondensation of trimethylhydroquinone with phytol or isophytol in the presence of an acid catalyst and organic solvent [13]. Commercial tocopheryl acetate is commonly used due to its availability and its stability under various conditions.

The use of synthetic vitamin E is necessary as there are very natural resources with high content of vitamin E. In order to increase the content of vitamin E in a cosmetic product, it would not make economic sense to pass the ingredients by a purification process as it will only add to the production cost. An answer to this dilemma (of trying to increases antioxidant content and keeping product prices low) is to substitute with ingredient that’s high in tocotrienols (sub-family of vitamin E). A previous study showed that tocotrienols have better antioxidant potency than tocopherols by a factor of 60 times [14]. Various studies have reported the benefit and potent effect of tocotrienols in dermatology especially in treating skin inflammation [9,15,16]. In this aspect, RPO stands out as a natural source of vitamin E which not only contains high tocopherols content (30% of total vitamin E) but also significant tocotrienol content (70% of total vitamin E) [2]. Around 90% of palm oil is applied for consumption and the benefit of RPO as edible oil has been widely documented. However, study on formulation and skin delivery of RPO, especially via nanoemulsion, has been scarce. Thus, this study was conducted to explore the potential of RPO for the preparation of nanoemulsions. The information will be useful for the industries that interested in developing pharmaceutical formulation or cosmetic products and the researchers in exploring the potential effect of RPO, especially on skin.

One challenging aspect of using RPO as a functional ingredient is its poor water solubility. McClements (2011) claimed that poor water solubility reduce the rate of absorption in the body, leading to low bioavailability[17]. One way to improve the limitations of the poor water solubility of red palm oil is to transform it from oil into oil-in-water nanoemulsions which can be used in cosmeceutical products. As generally accepted, the droplet size of nanoemulsionsusually less than 200nm, with 500nm being the upper limit—which is smaller than conventional emulsions [18–20]. A large numbers of literature had showed that, due to the small droplet size of nanoemulsions, they are kinetically stable and can withstand creaming, sedimentation, flocculation and coalescence. In other words, nanoemulsions are more stable than conventional emulsions [21–24]. Therefore, nanoemulsions play a role as a nano-carrier that can help in delivering active components to the target cells, endure instability and improving bioavailability in cosmeceuticals products [25,26].

Response Surface Methodology (RSM) is the most applicable multivariate statistical and mathematical tool for optimization analysis [27]. It evaluates optimum conditions of a research through a collection of mathematical and statistical techniques in the view on how well the data of the experiments fits in with a polynomial equation. One of the significant merits of RSM over a tradition optimization analysis (such as one-variable-at-a-time), is that it determines the interactions between the independent and dependent variables of a study with lesser number of experiments [28–30]. Therefore, it is less costly (reagents and materials) and time saving when compared to a tradition optimization analysis [27].

In this study, the process parameters for the preparation of tocotrienol-rich RPO-based nanoemulsion using high pressure homogenizer were optimized using Response Surface Methodology (RSM).

## Materials and methods

### Materials

RPO (Nutrolein, Goldren Palm Oil) was produced by UnitataBerhad (Perak, Malaysia). Polyoxyethylenesorbitan monooleate (Tween 80), sorbitan monooleate (Span 80) and glycerol was obtained from Fisher Scientific (Loughborough, United Kingdom), Merck (Darmstadt, Germany) and SystermChemAR (Shah Alam, Malaysia), respectively. The water used was Ultrapure water from Milli-Q Plus. For high-performance liquid chromatography (HPLC) analysis, isopropanol, hexane, methanol, acetonitrile, ethyl acetate, triethylamine and 1,4-dioxane were purchased from Fisher Scientific (Loughborough, United Kingdom). All chemicals and solvents used in the study were of analytical and HPLC grade.

### Determination of carotenoids and vitamin E

To extract the active ingredients in red palm oil, RPO, 1.0 mL of water and internal standard (t*rans* β-apo-8-carotenal for carotenoids analysis and 2,2,5,7,8-pentamethyl-6-chromanol for Vitamin E analysis) were added into centrifuge tube. The solution was vortexed for 1 minute. Then, 1.0 mL of isopropanol was added into the solution and vortexed for 1 minute. 10 mL of hexane then was added into the solution and vortexed for 2 minutes. The solution was centrifuged at 2500 rpm for 15 minutes at 4°C. After centrifuge, upper organic layer was drew out and transferred to a glass vial. Lastly, the transferred organic solution was dried with nitrogen gas. Oil extraction was done and dried samples were reconstituted with solvent diluents for HPLC analysis.

The carotenoids (Model LC-20AD, Shimadzu, Kyoto, Japan) and vitamin E (Model Prominence i, Shimadzu, Kyoto, Japan) in RPO were evaluated by HPLC following to a method described in[31,32], respectively with minor modification. For carotenoids analysis, isocratic mobile phase using a solvent mixture of methanol:acetonitrile:ethyl acetate (70:15:15, v/v/v, 0.1% v/v triethylamine) at 0.8 mL/min on a ODS Hypersil column (5 μm) (250 x 4.6 mm) (Thermo Scientific, Waltham, USA) resulted in an analysis time of 25 minutes, at 445nm and 450nm wavelength (UV-Vis detector). While for vitamin E, isocratic mobile phase using a solvent mixture of n-hexane: dioxane: isopropanol (97.5:2.0:0.5, v/v/v) at 1 mL/min on a Phenomenex Luna (Torrance, USA) 5 μm Silica C18 (2) 100 liquid chromatography column (250, 4.6 mm) resulted in an analysis time of 30 minutes, at 295nm (fluorescence detector excitation) and 325nm wavelength (fluorescence detector emission).

### Nanoemulsion preparation

Oil phase consisted of RPO and Span 80 while water phase consisted of Ultrapure water, glycerol, and Tween 80. Citric acid (0.08 wt%) was added into water phase as preservative. The oil phase was added to the water phase and premixed using high shear (Silverson L4R, Buckinghamshire, UK) at 6000 rpm for 10 minutes. The premixed mixtures then were passed through high pressure homogenizer (Panda 2 K, Niro Soavi, Deutschland, Lubeck, Germany).

### Droplet size and polydispersity index analysis

Droplet size and polydispersity index (PDI) of nanoemulsion was measured using Zetasizer Nano ZS (Worcestershire, U.K.) at 25°C. PDI is a measure of the droplet size distribution. It shows the percentage of droplets in distinct size classes.

### Selection of homogenization conditions and hydrophilic-lipophilic balance (HLB) value of mixed surfactant

Firstly, RPO nanoemulsion was prepared under different homogenization pressures (300, 600 and 900 bar) and cycles (one, four, seven and ten cycle). The nanoemulsions were prepared in the system of 5 wt% surfactant (Tween 80 only), 10 wt% glycerol, 20 wt% RPO and 65 wt% water for the homogenization determination. Based on droplet size, the most suitable homogenization pressure and cycle was chosen.

A range of HLB values 10–15 was used to prepare RPO nanoemulsion for the HLB value determination. In this study, nanoemulsion with 20 wt% RPO, 10 wt% glycerol and 60–65 wt% of water were tested with different HLB values (HLB:10–15; 5 & 10 wt%). The surfactant consists of the mixtures of Tween 80 and Span 80. Calculation of HLB values of mixed surfactants were calculated by the following formulation [33]:

$${\mathrm{HLB}}_{\mathrm{mix}}{=F}_{T}\;{\mathrm{HLB}}_{T}{\;+\;F}_{S}{\mathrm{HLB}}_{S},$$

Where HLB_mix_, HLB_T_, HLB_S_ are the HLB values of mixed surfactants, Tween 80 (T80, HLB = 15) and Span 80 (S80, HLB = 4.3), respectively, and F_T_, F_S_ are the weight fractions, of Tween 80 and Span 80.

The nanoemulsion was prepared under the same conditions (600 bar homogenization pressure, four cycles). RPO nanoemulsion was stored at 25°C for 35 days (five weeks) to assess the stability of the nanoemulsion. Droplet size of the RPO nanoemulsion was measured every week and the best HLB value was selected for this study.

### Transmission electron microscope analysis

The morphology of the red palm O/W nanoemulsion was analyzed by the transmission electron microscopy (TEM). The nanoemulsion was50 times diluted with deionized water. A droplet of diluted nanoemulsion was placed onto a 400-mesh formvar carbon film-coated copper grid. Then, the grid was negatively stained by uranyl acetate. The grid was left on a piece of Whatman filter paper to blot the excess liquid. After that, the grid was ready for analysis by JEM-2100F electron microscope (JOEL, Tokyo, Japan), operating at 200kV of accelerating voltage.

### Experimental design

RSM was used to optimize the condition for red palm O/W nanoemulsion. A three factor central composition design (CCD) analyzed the effect of manipulated variables surfactant concentration (X_1_:5–15 wt%), glycerol concentration in aqueous phase (X_2_:10–30 wt%) and homogenization pressure (X_3_: 500-700bar) on the droplet size (Y_1_) and PDI (Y_2_) of RPO nanoemulsion. The coded variables were shown in Table 1. A total of twenty randomized experiments were carried out, included six replicates of centre points.

**Table 1:** Coded levels of independent variables used in response surface methodology (RSM).

Independent variables	Symbols	Coded levels		
		-1	0	+1
Surfactant concentration (wt%)	X^1^	5	10	15
Glycerol concentration in aqueous phase (wt%)	X^2^	10	20	30
Homogenization pressure (bar)	X^3^	500	600	700

A second-order polynomial equation was used to predict the value of dependent variables (Y_1_& Y_2_) based on the study of independent variables (X_1_, X_2_& X_3_). The quadratic equation is as follows:

$$Y=\beta_{0}+\sum_{i=1}^{3}\beta_{i}X_{i}+\sum_{i=1}^{3}\beta_{ii}X_{i}^{2}+\sum \sum_{i<j=1}^{3}\beta_{ij}X_{i}X_{j}$$

Where, Y is the response (droplet size or PDI). β_0_, β_i_, β_ii_ and β_ij_ are the regression coefficients for intercept, linear, quadratic and interaction terms, respectively. X_i_ and X_j_ are the uncoded independent variables (surfactant concentration, glycerol concentration in aqueous phase and homogenization pressure).

### Verification of model

Optimized formulation of RPO nanoemulsion was generated by RSM under optimal conditions. Five experimental replicates were performed. The validity of the model was determined by comparing the experimental and predicted values.

### Statistical analysis

The Design Expert Version 6 software was used to carry out the experimental design and RSM analysis. SPSS was used for data analysis. Analysis of variance (ANOVA), paired-samples T test and Turkey’s post hoc test were applied to determine the significant differences among values under studied conditions.

## Results and discussion

### Carotenoids and vitamin E characterization

Red palm oil is one of the richest natural sources of carotenoids and tocotrienols. The carotenoids and vitamin E contents of RPO were identified in this study. The content of carotenoids and vitamin E in RPO are 664.994±1.946 ppm (0.44:0.56, α-carotene: β-carotene) and 999.485±20.023ppm, respectively (the dataset is available in S1 Dataset). These results were in line with other previous studies, which showed that, carotenoids content fell in the range of 500–700 ppm while vitamin E content was in the range of 500–1000 ppm[1–5].

The high content of carotenoids in RPO imparts the orange-red colour of RPO. While RBD palm oil contain no carotenoid as during the refining and deodorizing process, the carotenoids are removed and destroyed, therefore a light yellow RBD palm oil is produced. Choo and co-colleagues developed a process that can retain as much as 80% of the carotenoids of the crude palm oil in RPO[34]. In the market, carotenoids can be found in some vegetable oils, but the contents are low, generally less than 100 ppm [34]. Therefore, RPO is one of the natural richest sources of carotenoids. Its carotenoids (pro-vitamin A) equivalent content almost at 15 times that of carrots and 300 times that of tomatoes [4]

Tocopherols and tocotrienols are the two sub-members of vitamin E which in the form of α-, β-, γ- and δ-. Tocopherols and tocotrienols are different from their structure. Tocopherols molecules contain a long tail with no double bonds while tocotrienol molecules contain a short tails with three double bonds at C-3’, C-7’ and C-11’[35]. A comparison of the vitamin E isomers of RPO (data from this study) and other vegetables oil (data from previous studies) are shown in Table 2. Approximately 70% of the vitamin E in RPOis tocotrienols and 30% is tocopherols. In all of these vegetable oils, they are good source of vitamin E but mostly not a good source of tocotrienols. The tocotrienols content was significantly higher in RPO than the other vegetable oils.

**Table 2:** Total vitamin E (tocopherols and tocotrienols) content of RPO and other vegetable oils.

Products	Tocopherol (ppm)	Tocotrienol (ppm)	Total (ppm)
Red palm oil	255.342	744.143	999.485
RBD palm oil [36]	139	422	561
Soybean[37]	875.9	26.4	902.3
Corn Oil[37]	781.3	0	781.3
Olive Oil[37]	175.6	0	175.6
Sunflower Oil[37]	539.2	8.3	547.5
Wheat germ oil [37]	336.8	365.9	702.7

### Selection of homogenization conditions

**Fig 1:**
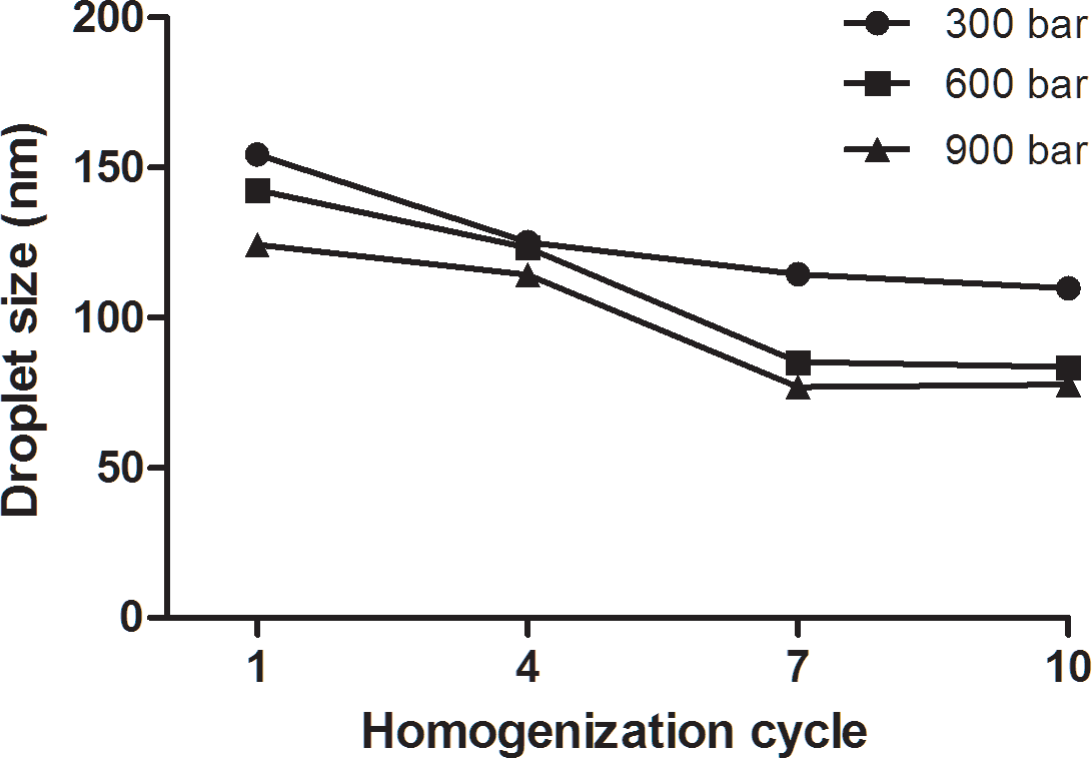
The effect of homogenization pressure and cycle on droplet size of nanoemulsion at 20 wt% of red palm oil (RPO), 5wt% of surfactant (Tween 80 only), 10 wt% of glycerol and 65wt% water (n = 3).

As shown in Fig 1, at any number of homogenization cycle applied, increasing the homogenization pressure (300–900 bar) resulted in significant decreases in droplet size. These findings were expected and agreed with the previous studies [38,39]. Significant change in droplet size of nanoemulsion could be interpreted by the shear forces and turbulence generated by the high pressure homogenizer [39,40]. The shear force and turbulence contribute to the breakage of the nanoemulsions into smaller droplets. The influence of homogenization cycle on the droplet size did show a consistent trend. The droplet size reached a plateau after a certain number of homogenization cycles. This implies that the energy supplied by high pressure homogenizer can reduce the size of droplets but after a certain number of cycles under the same pressure, the energy supplied by the homogenization cycles was insufficient to further reduce the droplet sizes, therefore a plateau was showed in the results [41].

In this study, homogenization pressure at 600 bar was selected for further investigation. This is due to the fact that a very low homogenization pressure (300 bar) resulted in a larger of droplet size as compared to 600 and 900 bar. Low homogenization pressure produce low turbulence effect which is insufficient to induce breakage of nanoemulsions during homogenization [21,40]. On the other hand, both homogenization pressure of 600 and 900 bar significantly (P<0.05) decreased droplet size of nanoemulsion. However, there is no significant difference (P>0.05) in droplet size at homogenization pressures of 600 and 900 bar after four, seven and ten homogenization cycles. This may be due to the interfacial layer that reach a saturation state [42,43]. At the maximum emulsification process, the surfactant was fully used to encapsulate oil for nanoemulsion formations, thus higher homogenization pressure (900 bar) had no significant effect on decreasing droplet size of the nanoemulsion. By manipulating system composition (oil and water composition and surfactant concentration), further decrease in droplet size may be observed [44].

Besides, at homogenization pressure of 900 bar, the temperature increased from 41°C at the first homogenization cycle to 53°C at the tenth cycle. The temperature of the nanoemulsion was increased due to the heat generated by the strong mechanical forces (frictional) of high-pressure-homogenizer during the process[45]. Thus, an increase of homogenization pressure and number of cycle are often accompanied by an increase of nanoemulsion temperature. Study showed that increased in the temperature of a sample during the high pressure homogenization affect the stability of nanoemulsion by influencing the properties of nanoemulsion (droplet size, viscosity and interfacial tension) [46]. Additionally, high homogenization pressure, homogenization cycle and temperature during processing can cause a degradation of properties such as vitamin [47]. Therefore, to minimize the loss of heat sensitive tocopherols, tocotrienols and carotenoids in RPO, 900 bar homogenization pressure and homogenization cycle more than four were not selected for further investigation. After studying the results, 600 bar homogenization pressure and 4 homogenization cycles were selected for the study.

### Selection of HLB value

One of the main driving forces for stable nanoemulsions is an appropriate HLB value of surfactant used in the formulation. It acts as a scale for selecting surfactants or combination of surfactants that required of the oil and water phase, especially mixed surfactants, as it reflects the ideal blend of surfactants that can leads to a stable nanoemulsion[48,49]. Low HLB surfactants form water-in-oil emulsion while high HLB surfactants form oil-in-water emulsion. Moreover, this can also save costs and time as it reduces the number of experiments during the screening part of the experiment [50]. Literatures showed that surfactants that have HLB value greater than 10 generally stabilize oil-in-water nanoemulsions[24,51,52]. Therefore, mixed surfactants with HLB values in the range 10 to 15 were studied in this study. In this study, the range of droplet size for 5 wt% and 10 wt% of surfactant were 96.47–130.90nm and 88.95–112.20nm respectively, which fulfilled the criteria of nanoemulsion (less than 200nm). The effects of HLB mixed surfactant on droplet size of the nanoemulsion at the day 0 and day 35 stored at 25°C were presented in Table 3. Among the HLB values, HLB 10 has the smallest nanoemulsion droplet size at 5 wt% (96.47nm) and 10 wt% (88.95nm) surfactant concentration. Meanwhile the biggest nanoemulsion droplet size was formed using HLB 14 (130.90nm) at 5 wt% surfactant and HLB 15 (112.20nm) at 10 wt% surfactant concentration.

**Table 3:** The effect of 5 wt% and10 wt% mixed surfactant with different hydrophilic-lipophilic balance (HLB) value on droplet.

	Droplet size (nm)			
	5 wt% of surfactant		10 wt% of surfactant	
HLB	Day 0	Day 35	Day 0	Day 35
10	101.85±4.20	123.13±0.45	88.95±0.55^c^	89.60±2.09^c^
11	127.40±1.40^a^	129.40±1.04^a^	98.92±1.03^d^	97.92±1.64^d^
12	115.47±1.67	137.57±1.17	102.57±2.30^e^	102.80±0.56^e^
13	122.63±0.40	125.73±0.57	110.80±1.93	106.93±1.60
14	130.87±1.95^b^	128.07±0.58^b^	101.57±1.98 ^f^	101.13±0.76 ^f^
15	120.93±1.46	124.90±0.79	112.17±1.57	129.47±1.01

The nanoemulsions were prepared in the system mixed surfactant/glycerol/RPO/water at 5–10 wt%, 10 wt%, 20 wt% and 65–60 wt% and at the homogenization pressure and cycle of 600 bar and 4 cycles, respectively. Values represent means and standard deviation (n = 3).

Same alphabets (a, b, c, d, e & f) showed no significant difference (P>0.05). The dataset is available in S3 Dataset.

Note: RPO, red palm oil.

Minimum droplet size with maximum stability is the goal of nanoemulsion formation, therefore stability of nanoemulsion was measured. In this study, nanoemulsion was stored at 25°C for 35 days and droplet size of nanoemulsion was measured every weeks. Among the HLB values, HLB 13 and HLB 15 were unstable at both surfactant concentrations, as their droplet size changed significantly after 35 days of storage. While 10 wt% of HLB 15 (T80:S80, 1:0) showed the most significant changes in droplet size after 35 days of storage, which had an increase droplet size up to 15.43%. Our results also show that mixed surfactants achieved better performance in term of nanoemulsion storage stability than pure surfactants (HLB 15), which is in agreement with the previous studies [22,53,54]. Studies found that the difference in headgroup-sizes of surfactants can influence the synergistic effects of mixed surfactants[55,56]. Larger headgroup-size differences contributed to larger synergistic effects, as small molecule surfactants can pack well with large surfactant at interface between oil and water phase. Thus, significance difference within the headgroup-size of Tween 80 and Span 80, promote synergistic effect between them which enhanced the stability of nanoemulsion systems [56]. Moreover, mixed surfactants enhanced the stability of nanoemulsion by reinforcing the interfacial film of nanoemulsion[57,58]. This might be due to the fact that mixed surfactants possess hydrophilic and lipophilic characters that favoured the adsorption between oil and water phase. Thus, mixed surfactants have better dispersity and solubility in continuous phase that can mix well with water and oil [53,59].

**Fig 2:**
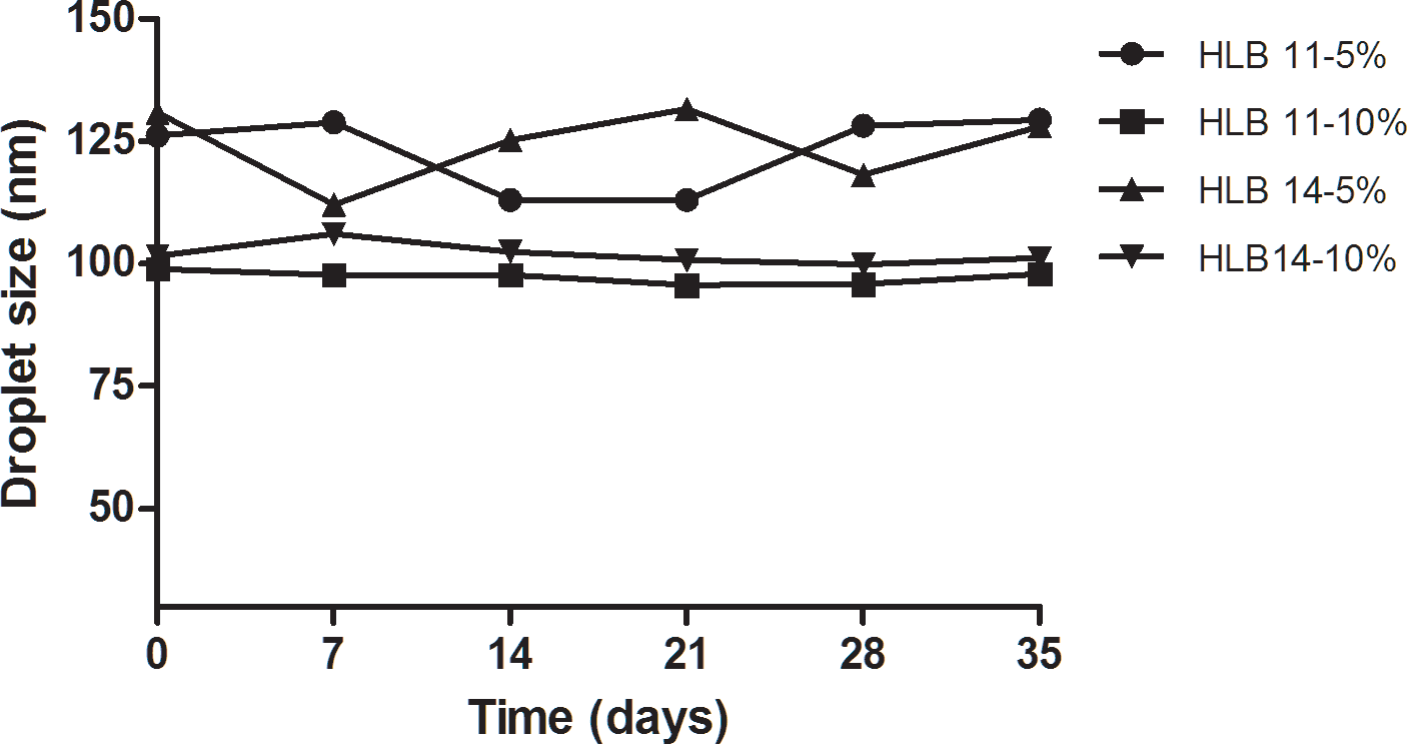
The effect of 5 wt% and 10 wt% of mixed surfactant (HLB 11& 14) on droplet size at 25°C storage temperature. The nanoemulsions were prepared at 20 wt% oil, 10 wt% glycerol, 60–65 wt% water, while 5 wt% and 10 wt% total mixed surfactant, at the homogenization pressure and cycle of 600 bar and 4 cycles, respectively.

On the other hand, nanoemulsion with HLB 11 (T80:S80, 0.63:0.37) and HLB 14 (T80:S80, 0.91:0.09) showed no significant difference in droplet size after 35 days of storage. However, Fig 2 shows HLB 11 was more stable than HLB 14 which indicated that HLB 11 was the most stable HLB value among others. One possible explanation is higher percentage of Span 80 (lipophilic) is needed to complete the surface coverage of nanoemulsion, in order to stabilize the 20 wt% RPO nanoemulsion. Small-sized nanoemulsion have larger surface area, thereby higher ratio of lipophilic surfactant (Span 80) is needed to cope with the greater adsorption of oil phase in nanoemulsion[60]. Our results was in agreement with the study of McClements, which showed that a range of HLB value within 10–12 is suitable to be used for O/W nanoemulsion, where it minimizes droplet size and enhances stability of nanoemulsion[24]. This is because surfactant at that range tends to be hydrophilic which favoured the formation and stability of O/W nanoemulsion. Moreover, the surfactant is surface active but do not affect the interfacial tension significantly [24]. A comparison between different types of surfactant was done by Rebolleda et al. [61]. They found that the droplet size of mixed surfactant (T80 and S80, HLB 11) was smaller than pure surfactant (single surfactant), Tween 20 (HLB = 16.7) and Tween 80 (HLB = 15). The results also showed that the mixed surfactant (T80 and S80, HLB 11) nanoemulsion was stable, because there was no significant difference in droplet size after 60 days storage at 4°C [61]. This is similar to our current works, RPO nanoemulsion prepared by 5 wt% and 10 wt% of mixed surfactant (HLB 11) were stable, as a result, there is no significant change in droplet size observed after 35 days of storage at 25°C. From the results given, HLB 11 was stable and able to produce small droplet size, therefore it was chosen to be used in this current study.

### Fitting the models

**Fig 3:**
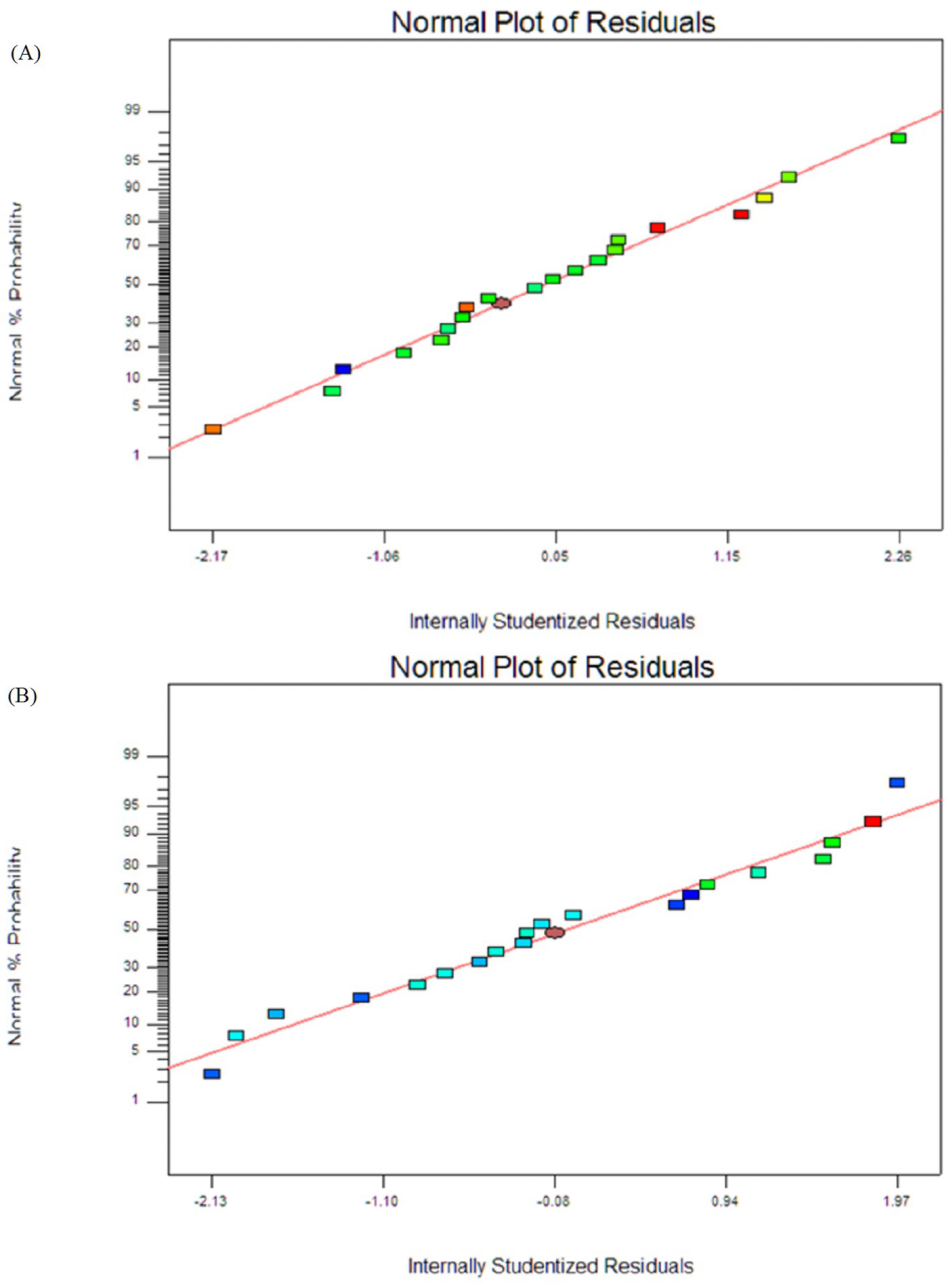
Normal probability plot residual for droplet size (A) & polydispersity index (B).

Table 4 shows the experimental and predicted value of droplet size and PDI of nanoemulsion corresponding to three independent variables. It shows that quadratic polynomial equations fitted the experimental data as indicated in Table 4. The coefficients of multiple determinations (R^2^) for particle size and PDI values were being 0.9115 and 0.8035, respectively. These readings indicated that the model could be predicted up to 91.15% of the droplet size observed value and 80.35% of the PDI observed values, which were high and led a high correlation between experimental data and the predicted values. The signal to noise ratio was measured by “Adep Precision” and a value more than 4 is a requirement for a good fit model[62,63]. The “Adep Precision” of responses droplet size and PDI were 13.41 and 9.942, respectively, indicates that the model is appropriate. The analysis of variance for both responses shows the model’s p-value is less than 0.05, indicates that the model is significant at 95% of probability level. Besides, the p-value of lack of fit is more than 0.05 which indicates no significant was found in droplet size and PDI response factors. Therefore, the quadratic model obtained was fitted with the data for the responses. Normal probability plot residuals for droplet size and PDI are shown in Fig 3A and 3B, respectively. The findings obtained mostly fall on a straight line, indicating that findings obtained are normally distributed and better matched with the regression model.

**Table 4:** Matrix of the central composite design (CCD) in coded levels, actualvalue and predicted value.

	Independent variables			Response variable			
Run	Surfactant amount, X_1_(wt%)	Glycerol concentration in aqueous phase, X_2_ (wt%)	Homogenization pressure, X_3_ (bar)	Droplet size (nm)		Polydispersity index	
				Actual value	Predicted value	Actual value	Predicted value
1	0	0	1	93.04	91.80	0.346	0.347
2	-1	1	1	102.30	98.48	0.301	0.326
3	-1	-1	1	89.23	90.84	0.390	0.402
4	1	0	0	96.52	87.79	0.340	0.351
5	0	0	-1	123.47	118.66	0.294	0.277
6	0	0	0	93.57	98.32	0.330	0.330
7	1	1	1	67.72	70.98	0.496	0.468
8	0	-1	0	108.40	103.01	0.342	0.345
9	0	0	0	100.50	98.32	0.320	0.330
10	-1	1	-1	118.10	119.40	0.281	0.232
11	0	1	0	93.59	92.93	0.382	0.361
12	1	-1	-1	117.00	122.34	0.298	0.270
13	-1	0	0	98.20	100.87	0.350	0.322
14	0	0	0	91.22	98.32	0.335	0.330
15	1	-1	1	89.32	89.54	0.344	0.316
16	0	0	0	96.37	98.32	0.375	0.330
17	0	0	0	95.50	98.32	0.327	0.330
18	1	1	-1	94.60	94.54	0.319	0.374
19	0	0	0	100.62	98.32	0.301	0.330
20	-1	-1	-1	122.74	121.00	0.334	0.356

Note: RPO, red palm oil; HLB, hydrophilic-lipophilic balance.

Table 5 also shows the significance of the coefficients of the regression model as determined by using analysis of variance (ANOVA). A big value of F-value and a small of P-value would imply a stronger significant influence on the respective response variables[62]. ANOVA of the regression coefficients of the quadratic equations for droplet size shows the linear term of homogenization pressure had the greatest effect on the droplet size of the nanoemulsion, followed by the linear term of surfactant concentration. The interaction between surfactant and glycerol concentration in aqueous phase also had a significant effect (P<0.05) on the droplet size of the nanoemulsion, while the other two interaction terms were insignificant (P>0.05). Moreover, none of the quadratic terms had a significant effect on the droplet size. On the other hand, ANOVA of the regression coefficients of the quadratic equations for PDI shows linear term of homogenization pressure and interaction between concentration of surfactant and concentration of glycerol in aqueous phase also had significant effect on size distribution. Linear term of surfactant concentration and glycerol concentration in aqueous phase showed no significant effect (P>0.05) on PDI. For quadric terms, the effect on PDI was shown to be insignificant (P>0.05).

**Table 5:** Analysis of variance of the regression coefficients of the quadratic equations for droplet size and polydispersity index (PDI) of red palm oil (RPO) nanoemulsion.

Variable	Droplet size (Y_1_)		PDI (Y_2_)	
	F-value	P-value	F-value	P-value
Model	11.45	0.0004	4.54	0.0135
Linear				
A_1_	14.59	0.0034	2.43	0.1498
A_2_	8.66	0.0147	0.62	0.4503
A_3_	61.48	<0.0001	15.08	0.0030
Quadric				
A_11_	1.49	0.2498	0.15	0.7026
A_22_	0.012	0.9167	1.90	0.1978
A_33_	4.47	0.0605	1.12	0.3151
Interaction				
A_12_	11.72	0.0065	15.19	0.0030
A_13_	0.12	0.7392	3.31	0.0990
A_23_	1.46	0.2545	1.38	0.2671
Lack of fit	3.18	0.1149	1.73	0.2806
R^2^	0.9115		0.8035	
Adep Precision	13.41		9.942	
2^nd^ order polynomial equation	98.32–6.54A-5.04B-13.43C-6.55AB-0.66AC+2.31BC-3.99A^2^-0.35B2+6.91C^2^		0.33+0.014A+7.100E-003B+0.035C+0.039AB+0.018AC+0.012BC+6.773E-003A^2^+0.024B^2^-0.018C^2^	

### Influence of independent factors on droplet size

**Fig 4:**
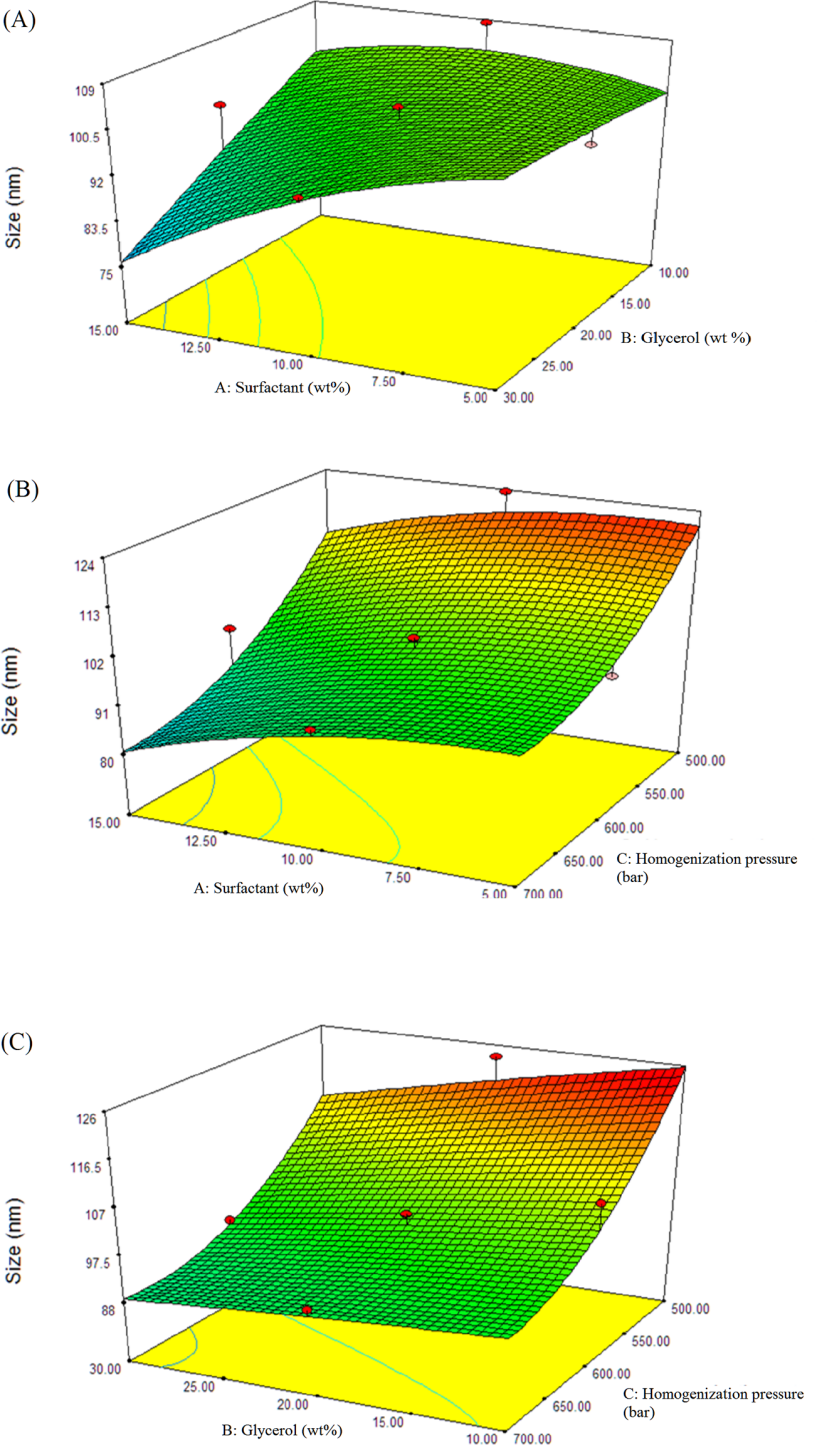
Effect of surfactant concentration, glycerol concentration in aqueous phase and homogenization pressure on droplet size of red palm oil (RPO) nanoemulsion. Response surface plot showing the effect of (A) 10 wt% of surfactant concentration and 20 wt% of glycerol concentration, (B) 10 wt% of surfactant concentration and 600 bar homogenization pressure, (C) 20 wt% of glycerol concentration and 600 bar homogenization pressure, on droplet size of red palm oil (RPO) nanoemulsion, missing independent variable in each figure was kept at the centre point.

Fig 4A shows a positive interactive effect, whereby droplet size of the nanoemulsion decreases when the surfactant concentration and glycerol concentration in aqueous phase increase. A significant decrease in nanoemulsion droplet size can be observed when the surfactant concentration is more than 7.5 wt% and glycerol concentration is more than 20 wt% in aqueous phase. The function of surfactant is to form a layer for adsorption of oil and water phase [39]. Thereby, the increase of surfactant concentration influences the oil and water mixture interfacial tension, results in decrease of droplet size[64]. Droplet break-up cause by the high pressure homogenization leads to smaller droplet size and new interfacial area[39]. These large and newly formed interfacial areas must be stabilized by surfactant. Therefore, a greater amount of surfactant is needed to cover the interaction between oil and aqueous of smaller size nanoemulsion. It also implies that, insufficient of surfactant to achieve adequate nanoemulsion coverage can be led to aggregation and coalescence of the nanoemulsion, thus larger droplet size [65,66]. It has been reported that the droplet size increased when decreasing concentration of surfactant [33,51].

Co-solvent such as glycerol has the capability to affect the formation and characteristic of nanoemulsions[67–69]. An addition of co-solvent can further decrease the droplet size due to the solubility of co-solvent which enhance the viscosity of aqueous phase, thus the dispersed-to-continuous phase viscosity ratio decrease and contribute to smaller droplet size [39,70]. Besides, co-solvent may influence the droplet size of nanoemulsion in the terms of solubility of surfactant. Co-solvent contained both hydrophilic and hydrophobic character that can penetrate into the monolayer of surfactant, therefore resulted a change of optimum curvature, interfacial tension and flexibility of surfactant [71–75]. This gives explanation on how the interactive effect between glycerol concentration and surfactant concentration had a significant effect on droplet size (P<0.05) in this study. The trend of decrease in droplet size when increase in concentration of surfactant and glycerol was also found when homogenization pressure was at 500 and 700 bar (figure was not shown, S1 Fig). This similar trend was also reported by Yuan and her co-workers’ works [60]. This probably means that increase of concentration of surfactant and glycerol resulted in the improvement of the size of nanoemulsion.

The interactive effect of surfactant concentration and homogenization pressure on the droplet size of nanoemulsion is shown in Fig 4B. At constant surfactant concentration, an increase of homogenization pressure led to a reduction of nanoemulsion droplet size. As expected, the shear forces and turbulence caused by the homogenization pressure can alter the properties of nanoemulsion (structure, stability, particles size, viscosity and interfacial tension) thus, resulted in decrease in droplet size of nanoemulsion[46]. A smaller droplet size of nanoemulsion was produced with conditions of higher percentage of surfactant and higher homogenization pressure [76,77]. The reduction in droplet size with the increase of homogenization pressure was in good agreement with the previous works [60,76,77]. A similar trend was also found on the interactive effect of glycerol concentration and homogenization pressure on droplet size of nanoemulsion, while surfactant concentration was kept constant (Fig 4C). This result shows homogenization pressure can significantly affect the droplet size of nanoemulsion but interactive effect with surfactant or glycerol concentration shows insignificant effect.

### Influence of independent factors on PDI

**Fig 5:**
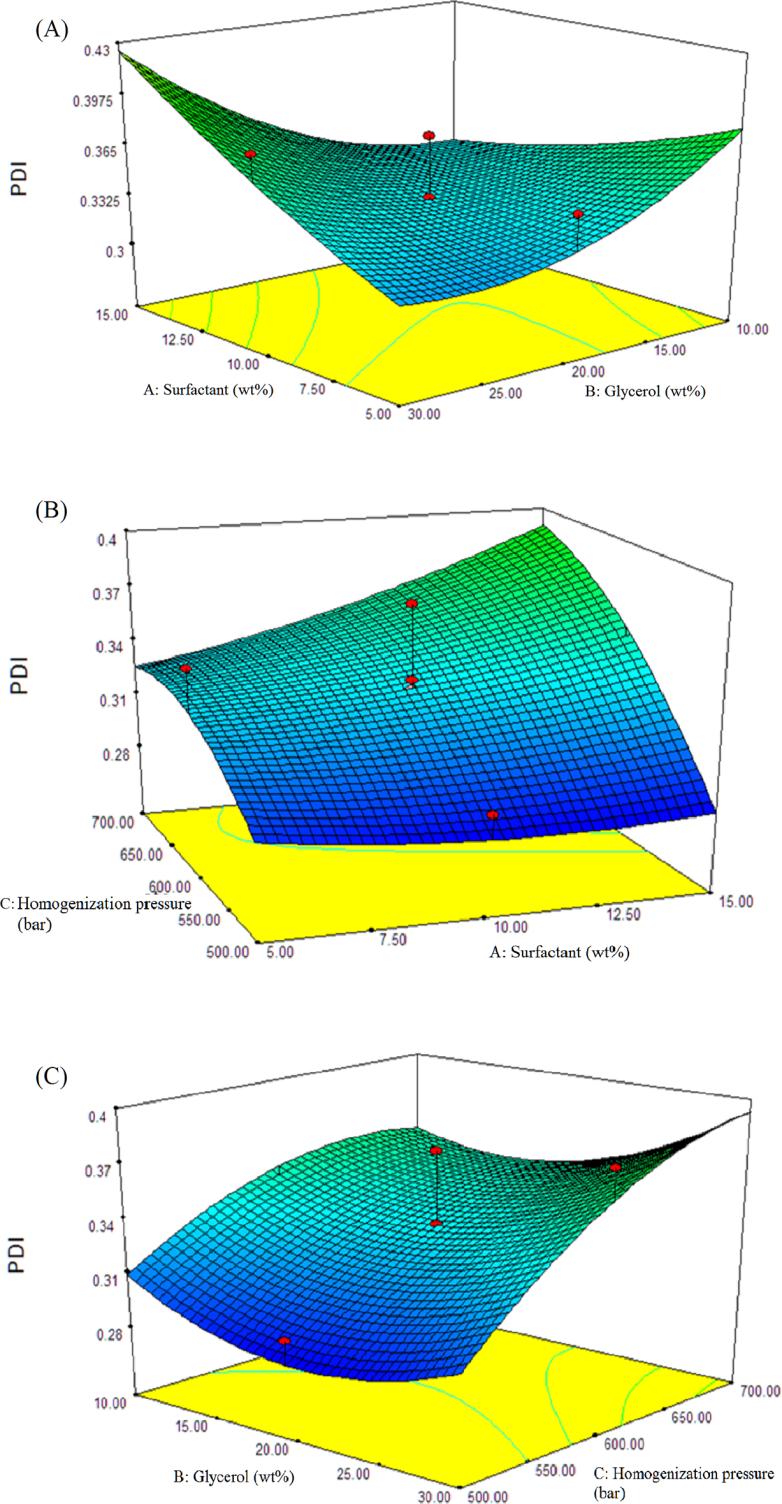
Effect of surfactant concentration, glycerol concentration in aqueous phase and homogenization pressure on polydispersity index of red palm oil (RPO) nanoemulsion. Response surface plot showing the effect of (A) 10 wt% of surfactant concentration and 20 wt% of glycerol concentration, (B) 10 wt% of surfactant concentration and 600 bar homogenization pressure, (C) 20 wt% of glycerol concentration and 600 bar homogenization pressure, on polydispersity index (PDI) of red palm oil (RPO) nanoemulsion, missing independent variable in each figure was kept at the centre point.

Polydispersity index is used as a factor to determine the stability of nanoemulsion[78]. It shows the homogeneity of the nanoemulsion[79]. Low value of PDI signifies high kinetic stability while high PDI has wider range of size and low stability. It was observed that, combination of low surfactant concentration (~<8 wt%) with low glycerol concentration (~<13 wt%) or combination of high surfactant concentration (>10 wt%) and higher glycerol concentration (>20 wt%), led to large PDI value (Fig 5A). A high PDI value may be due to a bimodal distribution developed or a monomodal distribution broadened during the homogenization [80]. This broadening droplet size distribution attributed by the insufficient or excess of surfactant and glycerol concentration. Studies found that in the presence of a high amount of undissolved nanoemulsions droplets due to insufficient of surfactant and glycerol will cause coalescence, therefore lead to larger droplet size and broadening the droplet size distribution [81,82]. Meanwhile, study also showed that PDI value increased with increasing surfactant concentration [80]. This might be due to the fact that when oil droplets are fully covered with surfactant, the surfactant and glycerol available increase, limiting the stabilization of the nanoemulsion system, thus increases droplet size distribution. Therefore, insufficient or excess of surfactant and glycerol concentration can destabilize the emulsification system and lead to high value of PDI.

In general, according to the Fig 5B and 5C, increase in homogenization pressure was unfavourable to the formation of nanoemulsion. We expected that increase homogenization pressure would contribute to smaller PDI, but these present results were in contrast. Based on the results, increase in homogenization pressure did not show a decrease of PDI in the interactive effect with surfactant and glycerol concentration. It showed that, at constant concentration of surfactant or glycerol, it led to increase of PDI value. Moreover, low PDI value was found at lower homogenization pressure, at any point of the surfactant and glycerol concentration. An increase of nanoemulsion PDI value in response to an increase of homogenization pressure has been reported in the literatures [47,83–85]. This can be explained by higher energy input during the homogenization that led to higher risk of “over-processing”. This phenomenon could be due to the fact that there was insufficient residue time for the surfactant and co-solvent to perform adsorption process on the surface of droplet before collisions between droplets [44]. Therefore, this resulted in bigger droplet size and wider range of different size classes. Besides, Floury, Desrumaux & Lardieresalso explained that at high homogenization pressure, recoalescence of the droplets during or after the homogenization process may have caused the wider size distribution[40]. Force applied by homogenization pressure led to the collision and breakdown of the interfacial membranes of nanoemulsion, then may affect the size distribution [24]. The explanation was in agreement with Mohan and Narsimhan, which reported that the stronger the turbulent force, the higher the collision rate between droplets, which increased the rate of coalescence[86]. Besides, there is no linear relationship between the decrease in PDI and reinforcement of the homogenization pressure and this observation is in line with the previous study [87].

## Optimization of conditions for preparing red palm oil-in-water nanoemulsion

The optimal conditions for the emulsification of the red palm O/W nanoemulsion used in this study would result in stable nanoemulsion with minimum droplet size and PDI. It would be preferable to have the droplet size and PDI as low as possible while the conditions of surfactant concentration, glycerol concentration in aqueous phase and homogenization rate as low as possible, in order to save cost. Therefore, 119.49nm of droplet size and 0.286 of PDI can be achieved with 6.09 wt% of surfactant, 20 wt% of glycerol in aqueous phase and 500 bar of homogenization pressure.

The validation of the experiment was carried out and the results of the optimum conditions suggested by RSM are displayed in Table 6 (the dataset is available in S5 Dataset). It can be observed that the droplet size and size distribution of the optimum conditions were in good agreement with the predicted value and was within 95% confidence interval.

**Table 6:** Comparison predicted value and observed value for droplet size and polydispersity index of red palm oil (RPO) nanoemulsion concentration.

Response variable (Y)	Predicted value with 95% confidence intervals	Observed value	Standard deviation
Droplet size (Y_1_)	111.93<120.82<129.7	119.49	9.624
PDI (Y_2_)	0.24<0.288<0.34	0.286	0.027

Note: PDI, polydispersity index.

## Transmission electron microscopic observation

**Fig 6:**
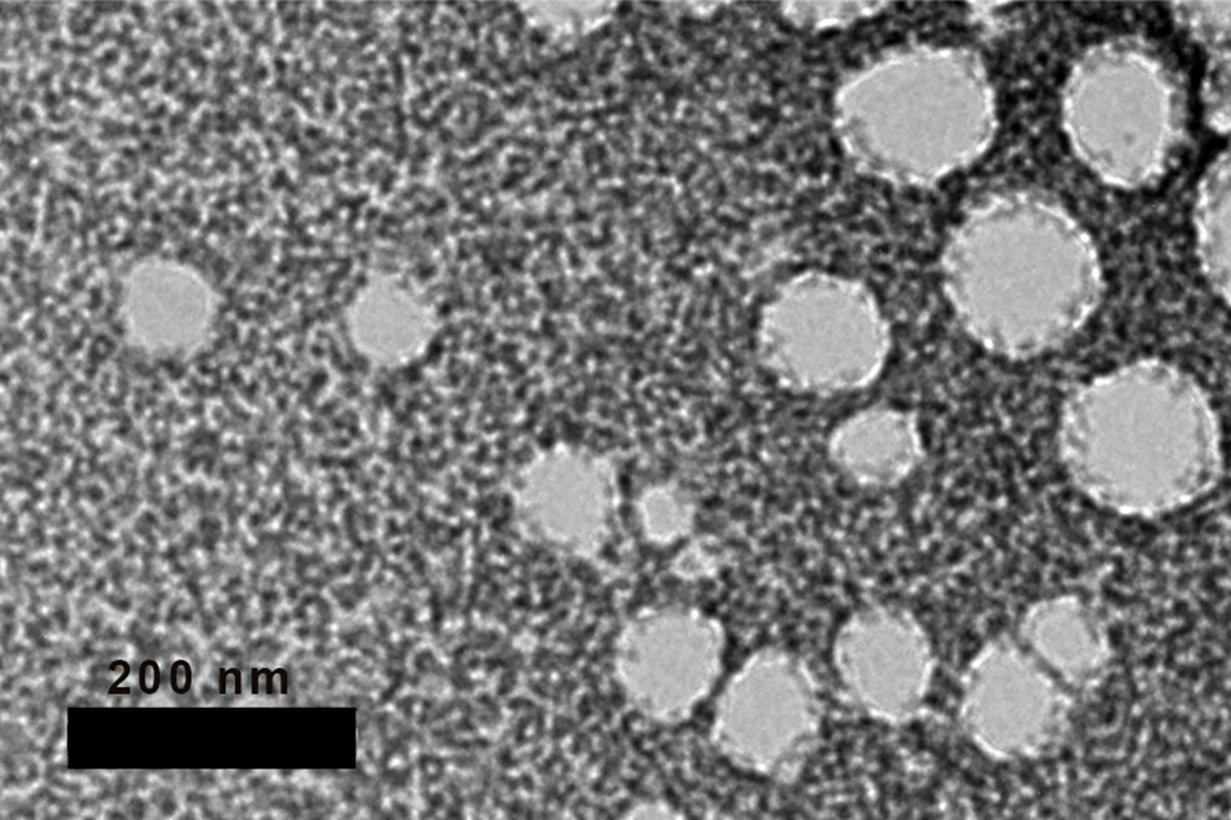
TEM image of red palm oil-in-water nanoemulsions. Scale bar represents 200nm.

Based on the Transmission electron microscopic (TEM) observations of the red palm O/W nanoemulsion, the shapes of nanoemulsion droplets were spherical (Fig 6).Droplet size of RPO nanoemulsion was less than 200nm. This agreed with the readings obtained from the verification of formulations (Table 6).

## Conclusion

CCD coupled to RSM was used in this study to determine optimum conditions of nanoemulsion, which led to the minimum size droplet and PDI. RSM demonstrated that the second-order polynomial model was sufficient to describe and predict the responses of the droplet size and PDI, with the change of emulsifying conditions (surfactant concentration, glycerol concentration in aqueous phase and homogenization pressure) within given experimental ranges. In general, linear term of homogenization pressure and interactive effect between surfactant and glycerol concentration were the significant factors that affected droplet size and PDI of red palm O/W nanoemulsion. The quadratic terms showed no significant influences in most cases. The observed value of the suggested optimum conditions was in agreement with the predicted value and within the 95% confidence interval. In the study, the model was proven effective to predict the optimum conditions of red palm O/W nanoemulsion. A small droplet size (119.69nm) and size distribution (0.286) of the nanoemulsion was successfully developed using 6.09 wt% of surfactant, 20 wt% of glycerol in aqueous phase and 500 bar of homogenization pressure. Thus, red palm O/W nanoemulsion with good water solubility and storage stability can be applied as natural source of vitamin E for skin-care and cosmoceutical products.

## Supporting information

S1 Dataset Readings of carotenoids and vitamin E content in Table 2. (DOCX)

S2 Dataset Readings of droplet size of nanoemulsion in Fig 1. (DOCX)

S3 Dataset Readings of droplet size of nanoemulsion in Table 3. (DOCX)

S4 Dataset Readings of droplet size of nanoemulsion in Fig 2. (DOCX)

S5 Dataset Readings of droplet size and PDI of nanoemulsion in Table 6. (DOCX)

S1 Fig The trend of decrease in droplet size when increase in concentration of surfactant and glycerol was also found when homogenization pressure was at 500 and 700. Response surface plot showing the effect of 10 wt% of surfactant concentration, 20 wt% of glycerol concentration and (A) 500 bar homogenization pressure and (B) 700 bar homogenization pressure.
(DOCX)
