# Supplementary material for: Optimization of process parameters in preparation of tocotrienol-rich red palm oil-based nanoemulsion stabilized by Tween80-Span 80 using response surface methodology
Source: PLoS One. 2018 Aug 24;13(8):e0202771. doi: 10.1371/journal.pone.0202771 (PMC6108518; doi:10.1371/journal.pone.0202771)
Supplement: S1 Dataset — (DOCX) [file pone.0202771.s001.docx]

**S1 Dataset. Readings of carotenoids and vitamin E content in Table 2.**

|  | Replicate 1 | Replicate 2 | Replicate 3 | Average | SD |
| --- | --- | --- | --- | --- | --- |
| RPO | mg/kg of oil | mg/kg of oil | mg/kg of oil | mg/kg of oil | mg/kg of oil |
| alpha-tocopherol | 250.648 | 258.366 | 257.012 | 255.342 | 4.121 |
| alpha-tocotrienol | 244.775 | 252.663 | 251.164 | 249.534 | 4.189 |
| beta-tocotrienol | 23.856 | 25.090 | 24.781 | 24.576 | 0.642 |
| gamma-tocotrienol | 381.029 | 387.657 | 386.819 | 385.168 | 3.609 |
| delta-tocotrienol | 76.654 | 91.496 | 86.445 | 84.865 | 7.546 |
| Total (ppm) | 976.962 | 1015.272 | 1006.221 | 999.485 | 20.023 |
|  |  |  |  |  |  |
|  |  |  |  |  |  |
|  | Replicate 1 | Replicate 2 | Replicate 3 | Average | SD |
| RPO | mg/kg of oil | mg/kg of oil | mg/kg of oil | mg/kg of oil | mg/kg of oil |
| *cis* alpha-carotene | 51.189 | 50.737 | 51.497 | 51.141 | 0.382 |
| *trans* alpha-carotene | 243.931 | 243.376 | 244.571 | 243.959 | 0.598 |
| beta-carotene | 370.009 | 368.871 | 370.800 | 369.893 | 0.970 |
| Total (ppm) | 665.129 | 662.984 | 666.869 | 664.994 | 1.946 |
