# Supplementary material for: Optimization of process parameters in preparation of tocotrienol-rich red palm oil-based nanoemulsion stabilized by Tween80-Span 80 using response surface methodology
Source: PLoS One. 2018 Aug 24;13(8):e0202771. doi: 10.1371/journal.pone.0202771 (PMC6108518; doi:10.1371/journal.pone.0202771)
Supplement: S2 Dataset — (DOCX) [file pone.0202771.s002.docx]

**S2 Database. Readings of droplet size of nanoemulsion in Fig 1.**

|  | Droplet size (nm) | | | |
| --- | --- | --- | --- | --- |
| Homogenization rate (bar)/ homogenization cycle | 1C | 4C | 7C | 10C |
| 300 | 153.8 | 126.7 | 111.9 | 108.5 |
|  | 155.7 | 124.4 | 113.3 | 111.1 |
|  | 153.6 | 124.1 | 117.9 | 109.6 |
|  |  |  |  |  |
| 600 | 143.2 | 121 | 84.72 | 82.83 |
|  | 144.3 | 122.9 | 85.34 | 83.9 |
|  | 140.3 | 125.7 | 85.67 | 84.19 |
|  |  |  |  |  |
| 900 | 123.3 | 119.1 | 73.87 | 75.9 |
|  | 126.9 | 115.2 | 74.22 | 81.68 |
|  | 122.5 | 120.4 | 82.63 | 76.46 |
