# Supplementary material for: Optimization of process parameters in preparation of tocotrienol-rich red palm oil-based nanoemulsion stabilized by Tween80-Span 80 using response surface methodology
Source: PLoS One. 2018 Aug 24;13(8):e0202771. doi: 10.1371/journal.pone.0202771 (PMC6108518; doi:10.1371/journal.pone.0202771)
Supplement: S3 Dataset — (DOCX) [file pone.0202771.s003.docx]

**S3 Dataset. Readings of droplet size of nanoemulsion in Table 3.**

| Percentage of Surfactant (%, w/w) |  | Day 0 | After 5 weeks |
| --- | --- | --- | --- |
|  |  | Droplet Size (nm) | Droplet size (nm) |
| 5 | HLB 10 | 106.3 | 123.1 |
|  |  | 101.3 | 123.6 |
|  |  | 97.95 | 122.7 |
|  |  |  |  |
| 10 | HLB 10 | 89.39 | 90.4 |
|  |  | 89.12 | 87.22 |
|  |  | 88.34 | 91.17 |
|  |  |  |  |
| 5 | HLB 11 | 125.8 | 128.7 |
|  |  | 128 | 128.9 |
|  |  | 128.4 | 130.6 |
|  |  |  |  |
| 10 | HLB 11 | 100.1 | 96.98 |
|  |  | 99.43 | 99.81 |
|  |  | 97.23 | 96.96 |
|  |  |  |  |
| 5 | HLB 12 | 113.6 | 137.8 |
|  |  | 116 | 138.6 |
|  |  | 116.8 | 136.3 |
|  |  |  |  |
| 10 | HLB 12 | 100.3 | 103.4 |
|  |  | 102.5 | 102.7 |
|  |  | 104.9 | 102.3 |
|  |  |  |  |
| 5 | HLB 13 | 122.4 | 125.1 |
|  |  | 123.1 | 126.2 |
|  |  | 122.4 | 125.9 |
|  |  |  |  |
| 10 | HLB 13 | 109.4 | 105.3 |
|  |  | 113 | 108.5 |
|  |  | 110 | 107 |
|  |  |  |  |
| 5 | HLB 14 | 131.4 | 128.0 |
|  |  | 128.7 | 128.8 |
|  |  | 132.5 | 127.4 |
|  |  |  |  |
| 10 | HLB 14 | 101.2 | 100.6 |
|  |  | 99.8 | 102 |
|  |  | 103.7 | 100.8 |
|  |  |  |  |
| 5 | HLB 15 | 119.3 | 124 |
|  |  | 121.4 | 125.2 |
|  |  | 122.1 | 125.5 |
|  |  |  |  |
| 10 | HLB 15 | 110.4 | 128.3 |
|  |  | 112.7 | 130 |
|  |  | 113.4 | 130.1 |
