# Supplementary material for: Optimization of process parameters in preparation of tocotrienol-rich red palm oil-based nanoemulsion stabilized by Tween80-Span 80 using response surface methodology
Source: PLoS One. 2018 Aug 24;13(8):e0202771. doi: 10.1371/journal.pone.0202771 (PMC6108518; doi:10.1371/journal.pone.0202771)
Supplement: S4 Dataset — (DOCX) [file pone.0202771.s004.docx]

**S4 Database. Readings of droplet size of nanoemulsion in Fig 2.**

|  | Droplet size (nm) | | | | | |
| --- | --- | --- | --- | --- | --- | --- |
| HLB-Surfactant % (w/w) | Day 0 | Week 1 | Week 2 | Week 3 | Week 4 | Week 5 |
| HLB 11-5% | 125.8 | 128.9 | 109.3 | 107.6 | 128.5 | 128.7 |
|  | 128 | 127.9 | 109.5 | 110.6 | 128.7 | 128.9 |
|  | 128.4 | 129.7 | 112.2 | 109.3 | 127.4 | 130.6 |
| HLB 11-10% | 100.1 | 97.27 | 97.64 | 94.52 | 95.24 | 96.98 |
|  | 98.43 | 98.14 | 97.89 | 96.28 | 96.5 | 99.81 |
|  | 98.23 | 97.93 | 97.79 | 96.28 | 95.75 | 96.96 |
| HLB 14-5% | 131.4 | 112 | 124.8 | 131.3 | 117.6 | 128.4 |
|  | 128.7 | 112.4 | 125.1 | 131.3 | 117.7 | 128.4 |
|  | 132.5 | 111.9 | 125.8 | 132.1 | 119.1 | 127.4 |
| HLB 14-10% | 101.2 | 105.8 | 102.9 | 101.2 | 98.54 | 100.6 |
|  | 99.8 | 104.5 | 101.7 | 100.7 | 100.9 | 102 |
|  | 103.7 | 107.7 | 102.7 | 100.3 | 100.1 | 100.8 |
