# Supplementary material for: Optimization of process parameters in preparation of tocotrienol-rich red palm oil-based nanoemulsion stabilized by Tween80-Span 80 using response surface methodology
Source: PLoS One. 2018 Aug 24;13(8):e0202771. doi: 10.1371/journal.pone.0202771 (PMC6108518; doi:10.1371/journal.pone.0202771)
Supplement: S5 Dataset — (DOCX) [file pone.0202771.s005.docx]

**S5 Dataset. Readings of droplet size and PDI of nanoemulsion in Table 6.**

| Droplet size (nm) | PDI |
| --- | --- |
| 114.9 | 0.268667 |
| 113.4333 | 0.270667 |
| 138.1333 | 0.289 |
| 112.1 | 0.308333 |
| 118.8667 | 0.295667 |
