# Supplementary material for: Optimization of process parameters in preparation of tocotrienol-rich red palm oil-based nanoemulsion stabilized by Tween80-Span 80 using response surface methodology
Source: PLoS One. 2018 Aug 24;13(8):e0202771. doi: 10.1371/journal.pone.0202771 (PMC6108518; doi:10.1371/journal.pone.0202771)
Supplement: S1 Fig — Response surface plot showing the effect of 10 wt% of surfactant concentration, 20 wt% of glycerol concentration and (A) 500 bar homogenization pressure and (B) 700 bar homogenization pressure. (DOCX) [file pone.0202771.s006.docx]

**S1 Fig. The trend of decrease in droplet size when increase in concentration of surfactant and glycerol was also found when homogenization pressure was at 500 and 700.**

(A)


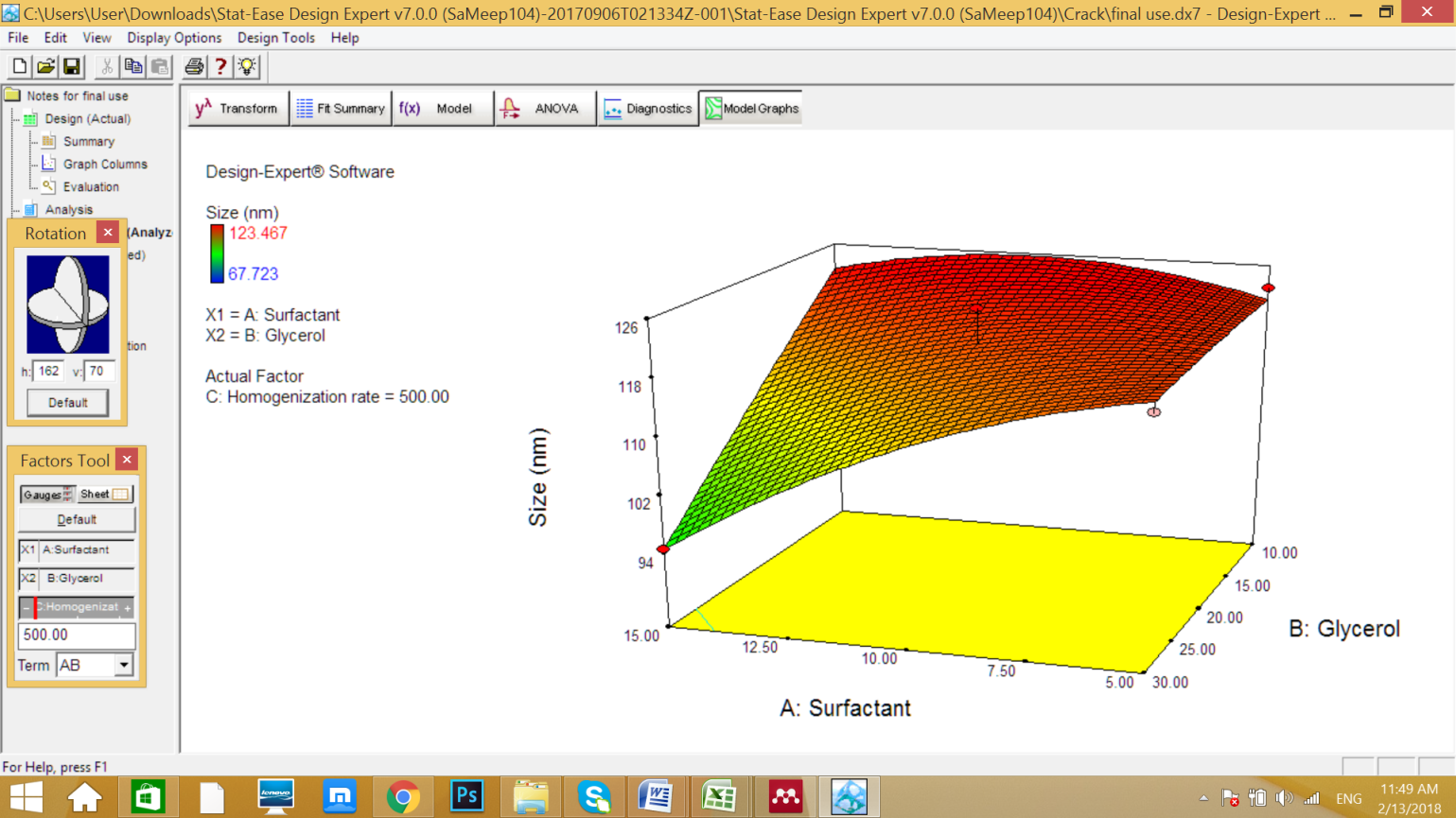
.

(B)

**
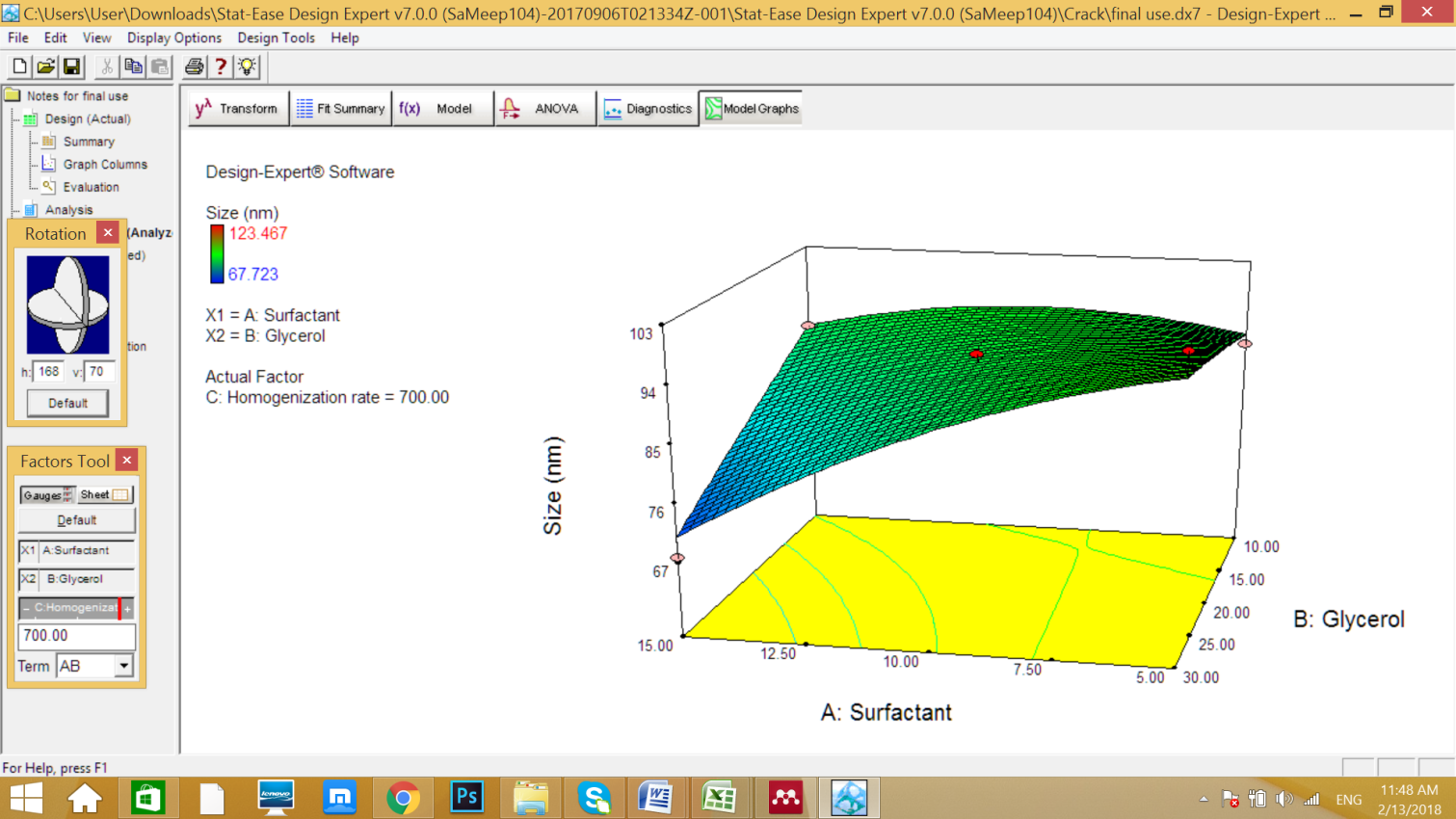
**

Response surface plot showing the effect of 10 wt% of surfactant concentration, 20 wt% of glycerol concentration and (A) 500 bar homogenization pressure and (B) 700 bar homogenization pressure
